# Evaluation of distribution of emerging mycotoxins in human tissues: applications of dispersive liquid–liquid microextraction and liquid chromatography-mass spectrometry

**DOI:** 10.1007/s00216-023-05040-8

**Published:** 2023-11-21

**Authors:** Ana Castell, Natalia Arroyo-Manzanares, Rosa Palma-Manrique, Natalia Campillo, Carmen Torres, José Fenoll, Pilar Viñas

**Affiliations:** 1 Department of Analytical Chemistry, Faculty of Chemistry, Regional Campus of International Excellence “Campus Mare Nostrum”, University of Murcia, E-30100 Murcia, Spain; 2 Department of Legal and Forensic Medicine, Faculty of Medicine, Biomedical Research Institute (IMIB-Arrixaca), University of Murcia, Murcia, Spain; 3 Murcia Institute of Agricultural and Alimentary Research and Development IMIDA, 30150 Murcia, Spain

**Keywords:** Emerging mycotoxins, Enniatins, Beauvericin, Bioaccumulation, Human tissues, DLLME

## Abstract

In this work, a complete study of the distribution of emerging mycotoxins in the human body has been carried out. Specifically, the presence of enniatins (A, A_1_, B, B_1_) and beauvericin has been monitored in brain, lung, kidney, fat, liver, and heart samples. A unique methodology based on solid–liquid extraction (SLE) followed by dispersive liquid–liquid microextraction (DLLME) was proposed for the six different matrices. Mycotoxin isolation was performed by adding ultrapure water, acetonitrile, and sodium chloride to the tissue sample for SLE, while the DLLME step was performed using chloroform as extraction solvent. Subsequently, the analysis was carried out by high-performance liquid chromatography coupled to tandem mass spectrometry (HPLC–MS/MS). The proposed method allowed limits of quantification (LOQs) to be obtained in a range of 0.001–0.150 ng g^−1^, depending on the tissue and mycotoxin. The precision was investigated intraday and interday, not exceeding of 9.8% of relative standard deviation. In addition, trueness studies achieved 75 to 115% at a mycotoxin concentration of 25 ng g^−1^ and from 82 to 118% at 5 ng g^−1^. The application of this methodology to 26 forensic autopsies demonstrated the bioaccumulation of emerging mycotoxins in the human body since all mycotoxins were detected in tissues. Enniatin B (ENNB) showed a high occurrence, being detected in 100% of liver (7 ± 13 ng g^−1^) and fat samples (0.2 ± 0.8 ng g^−1^). The lung had a high incidence of all emerging mycotoxins at low concentrations, while ENNB, ENNB_1_, and ENNA_1_ were not quantifiable in heart samples. Co-occurrence of mycotoxins was also investigated, and statistical tests were applied to evaluate the distribution of these mycotoxins in the human body.

**Graphical Abstract:**

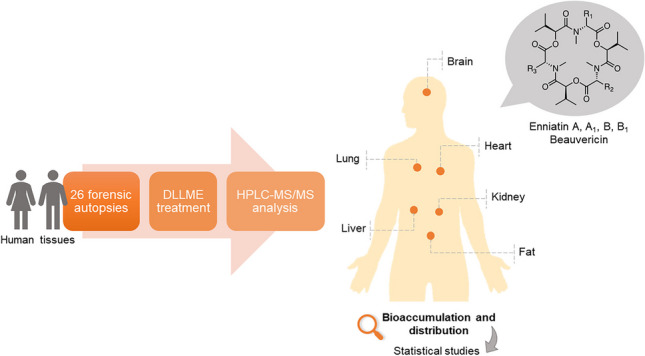

**Supplementary Information:**

The online version contains supplementary material available at 10.1007/s00216-023-05040-8.

## Introduction

Enniatins (ENNs) and beauvericins (BEAs), also known as emerging mycotoxins, are cyclodepsipeptides composed of three hydroxy acid groups and three N-methylamino acids biosynthesized by many species of *Fusarium* fungi as *F. acuminatum*, *F. avenaceum*, *F. poae*, or *F. oxysporum* [1]. These fungi grow on a wide variety of edible plants reaching significant concentrations. Moreover, this contamination is often non-homogeneous and localized which can spoil a whole batch of grains [2]. The occurrence of these secondary metabolites in feed and food stuffs has been extensively investigated, especially in cereals including rye, wheat, barley, and oats. Four enniatins (ENNB, A, B_1_, A_1_) and BEA are the most commonly detected mycotoxins [3].

The biosynthesis of ENNs and BEA in agricultural products is conditioned by multiple factors, such as the originating strain, the geographic and climatic conditions, or the different stages of the production process [4]. Crops contaminated with these mycotoxins can pose a risk to animal and human health by introducing them into the food chain. Some studies have linked the presence of ENNs and BEA to alterations in both male and female reproductive functions in animals. However, further studies are required to confirm the toxicity of these mycotoxins [5]. The Panel on Contaminants in the Food Chain (CONTAM) of the European Food Safety Authority (EFSA) stated that the acute exposure to ENNs and BEA does not constitute a hazard to human health and does not define an acute reference dose or a tolerable daily intake for these secondary metabolites. However, the panel suggests considering their chronic exposure due to the lack of conclusive data on their toxicity, which is still under study [6]. 

BEA has been related with anticancer, insecticidal, antimicrobial, nematocidal, and cytotoxic activity [7]. The toxicity of BEA is associated to its ion-transporting activity through the cytoplasmic membrane conferred by its molecular structure. Other activities of this mycotoxin are the inhibition of enzymes or the induction of oxidative stress [7]. ENNs exhibit insecticidal, herbicidal, antifungal, and antimicrobial properties and show high toxicity to mitochondria [2].

The determination of BEAs and ENNs in animals and humans has also been investigated. Liquid chromatography (LC) is widely used for the determination of these mycotoxins in biological matrices, combined with tandem mass spectrometry (MS/MS) or high-resolution mass spectrometry (HRMS), becoming the benchmark techniques nowadays. Regarding animal studies, plasma [8–13] is the most commonly used matrix for this purpose followed by urine [9, 14–16], feces [9, 14, 16]**,** and serum [14–16], mainly from pigs and broiler chickens. Other matrices as rumen fluid [17] as well as some tissues and organs from fish [18] and mice [15] have also been studied. In terms of human samples, urine is the most analyzed fluid [19–25], although plasma [22] and serum [26] have also been explored. The occurrence of these mycotoxins in human organs has only been investigated in liver, together with aflatoxins, HT-2 and T-2 toxins, deoxynivalenol, and zearalenone [27], concluding that ENNB was the most frequently detected mycotoxin in human samples. 

The development of sensitive and accurate analytical methods is essential to evaluate human exposure to emerging mycotoxins and to investigate their toxicokinetics. So far, these studies have focused on biological fluids, and studies on the bioaccumulation and distribution of these secondary metabolites in the human body by analysis of organs or tissues are scarce. Biological matrices are often complex and require effective sample treatments to isolate and preconcentrate the analytes. Analyte isolation and sample clean-up step based on deproteinization, in the case of plasma and serum, have been carried out mainly using acetonitrile (ACN) [9–13, 15, 16, 18] or ethyl acetate [9, 14, 17]. Other proposed procedures were QuEChERS extraction [19], salting out liquid–liquid extraction (SALLE) [25], or dispersive magnetic solid-phase extraction (DMSPE) using magnetic multiwalled carbon nanotubes [21] for the analysis of human urine. Miniaturized techniques such as dispersive liquid–liquid microextraction (DLLME) [13, 24, 27] have also been carried out as sample pre-treatment. This technique provides high enrichment factors and recoveries using lower organic solvent volumes and operating fast and easily [28]. 

In the present work, an analytical methodology based on DLLME followed by high-performance liquid chromatography coupled to MS/MS with triple quadrupole (QqQ) analysis is proposed for the determination of emerging mycotoxins in the human body. For the first time, six different tissues (brain, fat, kidney, liver, heart, and lung) obtained from 26 autopsies have been studied. The co-occurrence of these mycotoxins was investigated, and statistical studies were performed with the aim to evaluate the bioaccumulation and distribution of BEA, ENNA, B, A_1_, and B_1_ in the human body. 

## Materials and methods

### Standards and reagents

All solvents were of HPLC grade and reagents were of reagent grade. Methanol (MeOH) and ACN were supplied by ChemLab (Zedelgem, Belgium). Sodium chloride, formic acid, ammonium formate, and chloroform were obtained from Sigma-Aldrich (St. Louis, MO, USA). Individual standards of emerging mycotoxins were also purchased from Sigma-Aldrich. Solutions of each mycotoxin were prepared at 1000 μg mL^*−*1^ in ACN and stored at − 20 °C.

A Milli-Q system from Millipore (Bedford, MA, USA) was used to obtain the ultrapure water (18.2 mΩ cm^*−*1^).

### Tissue samples

Human tissues (liver, fat, kidney, heart, brain, and lung) were obtained from 26 autopsies at the Institute of Legal Medicine of Murcia (Spain) (about 8–10 g of each tissue). Samples were from men and women aged between 29 and 86 from suicide, accidental, or natural deaths. The specific data for each autopsy are shown in Table S1. A total of 152 samples was analyzed by the proposed methodology: 25 of liver and heart; 26 of kidney, lung, and brain; and 24 of fat. Human tissues were part of the regular tests for cause-of-death diagnosis and were not taken specifically for investigation. This study was developed in compliance with current legislation, respecting the personal data protection and according to the requirements of the ethical committee of the University of Murcia for this type of studies (University of Murcia, 1848/2018).

Lamb tissues were used for the optimization and validation of the method due to their similarity to human tissues and the limited availability of human forensic samples [29]. These animal tissues were purchased in local markets.

All tissues were milled to obtain homogeneous samples and individually stored at − 20 °C before analysis.

### Instrumentation and software

All experiments were performed using a 1200 high-performance liquid chromatograph combined with a 6410 triple quadrupole mass spectrometer from Agilent Technologies (Santa Clara, CA, USA). The system was equipped with an electrospray ionization (ESI) operating in positive mode. An InfinityLab Poroshell 120 EC-C18 column (4.6 $\times$ 100 mm $\times$ 2.7 µm) also from Agilent was used for chromatographic separation. Data were collected and processed using the MassHunter Quantitative Analysis (QqQ) software, using the multiple reaction monitoring (MRM) mode. SigmaPlot 13.1 (Systat Software, San Jose, CA, USA) and MetaboAnalyst 5.0 software were used for the statistical analysis.

An EBA 20 centrifuge (Hettich, Tuttlingen, Germany), an XcelVap air-drying system (Horizon Technology, Salem, MA, USA) and an LLG-uniTEXER vortex (Heathrow Scientific, IL, USA) were used during sample treatment. Tissue samples were ground with an IKA A11 basic mixer (Wilmington, USA). Nylon syringe filters of 0.45 μm $\times$ 25 mm and 0.2 μm $\times$ 13 mm from Agilent were used to filter the sample extracts.

### Sample treatment and HPLC-QqQ-MS/MS analysis

Sample treatment procedure was previously optimized for the determination of 13 mycotoxins in human and animal liver samples [27]. This procedure consisted of adding 3 mL of ultrapure water, 3 mL of ACN, and 1 g of sodium chloride to 3 g of tissue. After shaking and centrifuging at 4500 rpm (1924 *g*) for 5 min, 2 mL of the supernatant filtered (0.45 μm) was used for the DLLME extraction with 6 mL of ultrapure water and 600 µL of chloroform as extractant solvent. The sedimented phase obtained after centrifuging during 3 min at 3500 rpm (1164 *g*) was collected and dried using an airstream. The sample was reconstituted with MeOH:H_2_O (50:50 v/v) in 500 µL, filtered (0.2 μm), and injected into the system.

The chromatographic separation was carried out using a mobile phase composed of aqueous solution at 0.1% of formic acid with 2 mM of ammonium formate as solvent A, and MeOH with 0.1% of formic acid as solvent B. The sample volume injected into the system was 20 µL and the flow rate through chromatographic column was 0.5 mL min^−1^. The eluent gradient was optimized from the liver study for the separation of emerging mycotoxins at short elution times. This program was started at 80% B, reached 100% B after increasing linearly for 5 min (held for 5 min) and back to 80% B at 12 min. Emerging mycotoxins were determined using the MRM conditions shown in Table S2. The temperature ionization source was 350 °C, capillary voltage was 3000 V, nebulizer pressure was 40 psi, and the gas flow was set at 9 L min^−1^. Fragmentor voltage and collision energy (EC) were 130 and 30 eV, respectively.

## Results and discussion

### Optimization of the proposed method

The mobile phases of the chromatographic method were selected to provide a suitable analytical signal for the quantification of emerging mycotoxins. ENNs and BEAs are ionophore compounds that can form complexes with monovalent and divalent cations through weak interactions with the carbonyl groups oriented towards the center of the molecule. Due to their flexible structure, these mycotoxins are not very cation selective and different complexes can be formed during analysis (Na^+^, K^+^, Ca^2+^, and NH_4_^+^) [30]. Ammonium formate and formic acid were used in this work, which promote the formation of the [M + NH_4_]^+^ adduct [31]. In our experiments, both [M + H]^+^ and [M + NH_4_]^+^ cations were observed, being prevalent the [M + NH_4_]^+^ cation formation, and therefore this ion was used for mycotoxin quantification.

**Fig. 1:**
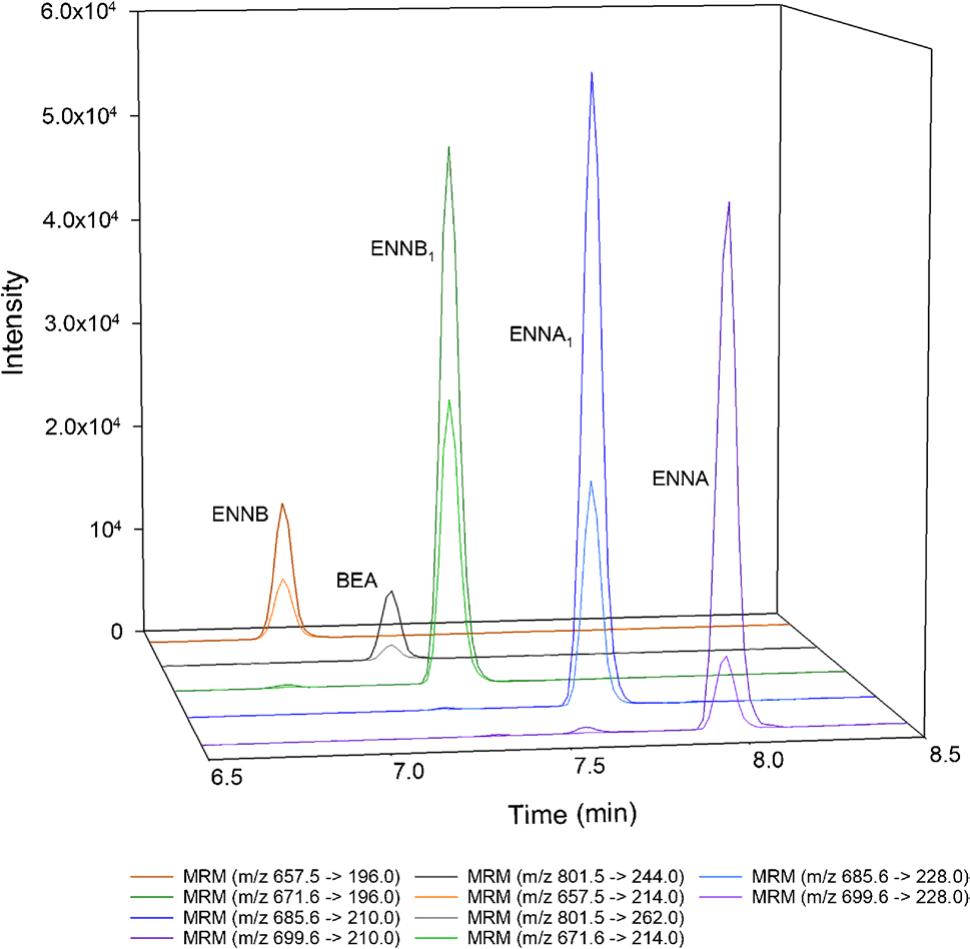
Chromatographic separation of mycotoxins in a lung sample spiked at 5 ng g^−^.^1^

The gradient program was adjusted to the elution time of ENNs and BEAs. The optimization was carried out using a standard mycotoxin solution prepared at 100 ng mL^−1^ in MeOH:H_2_O (50:50 v/v) under the conditions specified in the previous section. The following elution gradients were tested: 0–10 min: 30–99% B, 10–20 min: 99% B, 20–22 min: 99–30% B (gradient 1); 0–10 min: 70–100% B, 10–16 min: 100% B, 16–18 min: 100–70% B (gradient 2); and 0–5 min: 80–100% B, 5–10 min: 100% B, 10–12 min: 100–80% B (gradient 3). Finally, gradient 3 was selected, as it allowed adequate separation of the five mycotoxins in a shorter elution time. The total elution time was 12 min and the retention times obtained were between 6.8 and 8 min. The chromatographic separation of emerging mycotoxins using the proposed methodology in a lung sample spiked at 5 ng g^−1^ is shown in Fig. 1.

**Fig. 2:**
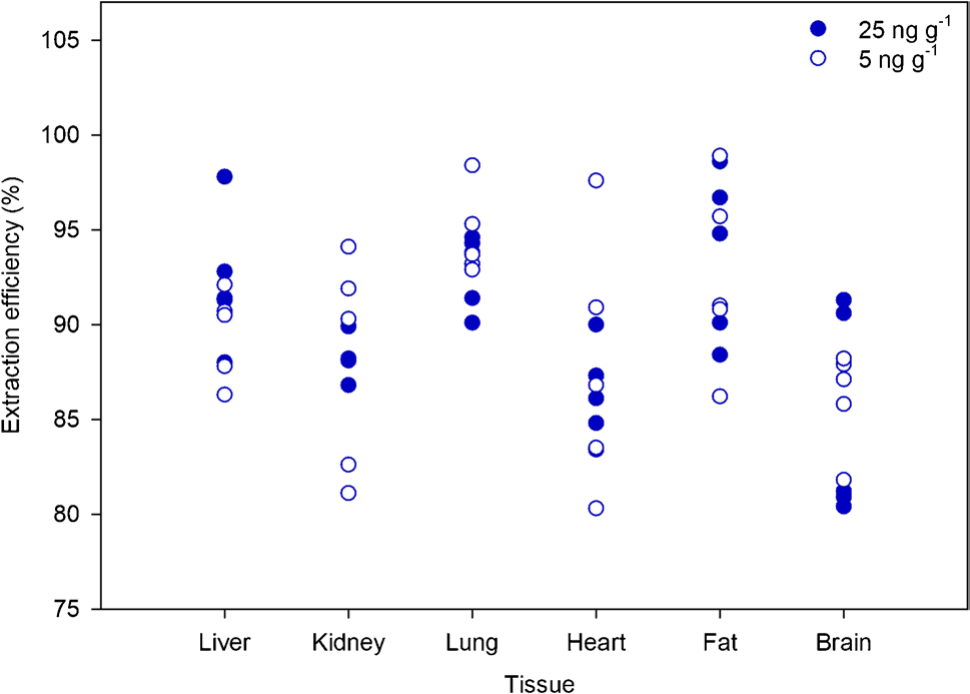
Extraction efficiency of emerging mycotoxins in the different tissues at 25 and 5 ng g^*−*^.^1^

The extraction efficiency of the proposed sample procedure was evaluated for all tissues investigated in this work (liver, fat, brain, lung, kidney, and heart). Thus, samples were spiked at two different concentrations, 25 and 5 ng g^*−*1^, before and after extraction, and were prepared in duplicate. Lamb tissues were used as reference matrix to perform these experiments. Extraction efficiency was calculated by comparing the analyte peak area o*btained when sample was spiked* after extraction *related to that obtained when sample was s*piked before extraction. Figure 2 shows the results for the five emerging mycotoxins at the two different concentrations. In all cases, extraction was higher than 80%, ranging up to 98.9%. Similar extraction efficiency values were achieved for all mycotoxins. Therefore, the proposed sample treatment based on DLLME was considered efficient for all matrices, which provides an advantage, as a unique and simple extraction can be applicable to six human tissues, which are often complex matrices to handle.

### In-house validation of the analytical method

The characterization of the proposed methodology was evaluated in terms of linear dynamic range, limit of detection (LOD) and quantification (LOQ), trueness, and precision, following the EU Commission Decision 96/23/EC [32]. Matrix effect was also assessed to check the influence of the different tissues on the mycotoxin determination. These experiments were also performed using lamb tissue samples. Tables 1 and 2 summarize the results achieved for each performance characteristic.

**Table 1:** Validation data of the proposed methodology

Tissue	Analyte	LR^a^ (ng g^−1^)	*R*^2^	LOD^b^ (ng g^−1^)	LOQ^c^ (ng g^−1^)	Matrix effect (%)	Trueness (% RSD^d^)	
							25 ng g^−1^	5 ng g^−1^
Brain	ENNB	0.031–50	0.997	0.009	0.031	24.5	96 (1.1)	115 (0.8)
	BEA	0.010–50	0.999	0.003	0.010	3.6	96 (1.5)	104 (1.5)
	ENNB_1_	0.039–50	0.999	0.012	0.039	− 1.5	94 (1.8)	93 (8.3)
	ENNA_1_	0.027–50	0.999	0.008	0.027	− 29.7	101 (0.3)	96 (5.2)
	ENNA	0.043–50	0.999	0.013	0.043	19.6	100 (1.7)	103 (1.9)
Liver	ENNB	0.036–50	0.998	0.011	0.036	− 14.3	94 (2.1)	101 (1.2)
	BEA	0.006–50	0.998	0.002	0.006	− 8.9	111 (9.7)	82 (1.0)
	ENNB_1_	0.027–50	0.999	0.008	0.027	− 15.9	84 (3.6)	103 (2.2)
	ENNA_1_	0.049–50	0.999	0.015	0.049	− 25.9	82 (4.0)	95 (1.2)
	ENNA	0.053–50	0.998	0.016	0.053	− 21.1	75 (4.6)	94 (5.5)
Lung	ENNB	0.001–50	0.999	0.0003	0.001	− 29.6	105 (2.1)	97 (5.6)
	BEA	0.001–50	0.999	0.0003	0.001	− 33.6	100 (8.2)	96 (0.5)
	ENNB_1_	0.015–50	0.999	0.005	0.015	− 38.4	105 (3.8)	93 (3.6)
	ENNA_1_	0.036–50	0.999	0.011	0.036	− 51.1	105 (4.4)	93 (5.4)
	ENNA	0.096–50	0.999	0.029	0.096	− 42.0	103 (4.2)	101 (8.4)
Kidney	ENNB	0.006–50	0.999	0.002	0.006	− 28.1	100 (6.4)	93 (3.3)
	BEA	0.036–50	0.999	0.011	0.036	− 14.3	115 (4.8)	108 (6.3)
	ENNB_1_	0.065–50	0.997	0.020	0.065	− 41.3	102 (2.5)	109 (4.7)
	ENNA_1_	0.150–50	0.999	0.045	0.150	− 33.9	107 (2.6)	99 (5.9)
	ENNA	0.127–50	0.998	0.038	0.127	23.0	109 (2.0)	97 (3.5)
Heart	ENNB	0.028–50	0.999	0.008	0.028	5.9	109 (3.8)	109 (5.9)
	BEA	0.002–50	0.998	0.0005	0.002	− 17.0	89 (7.2)	94 (2.7)
	ENNB_1_	0.069–50	0.998	0.021	0.069	− 5.6	118 (3.1)	107 (2.2)
	ENNA_1_	0.078–50	0.999	0.023	0.078	− 22.3	106 (2.0)	112 (2.7)
	ENNA	0.138–50	0.999	0.041	0.138	− 38.3	107 (5.9)	111 (2.4)
Fat	ENNB	0.015–50	0.997	0.005	0.015	27.6	110 (3.0)	87 (5.6)
	BEA	0.082–50	0.994	0.025	0.082	− 34.7	107 (5.4)	108 (3.1)
	ENNB_1_	0.063–50	0.995	0.019	0.063	− 1.2	112 (2.8)	118 (3.6)
	ENNA_1_	0.034–50	0.995	0.010	0.034	10.5	114 (3.8)	118 (3.6)
	ENNA	0.033–50	0.997	0.010	0.033	6.3	102 (3.9)	112 (3.8)

^a^*LR*, linear range

^b^*LOD*, limit of detection

^c^*LOQ*, limit of quantification

^d^*RSD*, relative standard deviation (*n* = 3)

**Table 2:** Precision results of the proposed methodology

Tissue	Analyte	Repeatability, % RSD (*n* = 6)		Intermediate precision, % RSD (*n* = 6)	
		25 ng g^−1^	5 ng g^−1^	25 ng g^−1^	5 ng g^−1^
Brain	ENNB	1.2	9.2	8.6	9.8
	BEA	1.5	6.4	4.8	7.0
	ENNB_1_	1.9	4.5	4.0	4.3
	ENNA_1_	0.3	5.7	3.8	5.6
	ENNA	1.7	2.0	6.5	6.4
Liver	ENNB	1.6	8.6	7.7	9.0
	BEA	8.5	8.4	1.0	1.3
	ENNB_1_	2.6	3.8	4.5	4.6
	ENNA_1_	1.1	7.2	3.3	9.2
	ENNA	2.7	5.2	3.0	4.3
Lung	ENNB	3.6	7.1	8.2	9.3
	BEA	5.8	6.4	7.1	8.7
	ENNB_1_	4.3	6.1	5.8	7.3
	ENNA_1_	5.6	2.9	6.0	7.7
	ENNA	2.9	5.6	5.7	6.5
Kidney	ENNB	1.9	5.4	8.3	7.9
	BEA	4.9	5.5	6.3	7.4
	ENNB_1_	7.5	6.2	7.8	7.6
	ENNA_1_	3.2	3.4	5.8	4.0
	ENNA	1.8	1.2	1.9	7.5
Heart	ENNB	4.8	6.0	8.0	9.7
	BEA	3.2	4.3	3.3	3.0
	ENNB_1_	3.5	6.7	1.9	9.8
	ENNA_1_	5.1	6.5	4.0	8.0
	ENNA	1.9	5.3	6.3	6.9
Fat	ENNB	2.9	6.2	4.4	8.3
	BEA	5.7	5.2	9.2	9.3
	ENNB_1_	2.7	5.1	1.8	4.1
	ENNA_1_	3.7	5.2	4.5	4.9
	ENNA	3.8	1.5	4.9	9.7

*RSD*, relative standard deviation

Matrix effect was studied by spiking the sample at 25 ng g^*−*1^ of each mycotoxin and calculated by [(analyte signal in spiked sample − analyte signal in standard solution) / analyte signal in standard solution] × 100. The results showed a significant suppression or enhancement response in the determination of some mycotoxins in certain tissues, especially in lung samples, showing up to a 50% of signal suppression for ENNA. The presence of matrix effect may affect the performance of the proposed method, so matrix-matched calibration curves were performed for the accurate quantification. Thus, each tissue was spiked at five concentrations (0.5, 2, 5, 25, and 50 ng g^*−*1^) of ENNs and BEAs. All samples were prepared in duplicate. Mycotoxins showed a good linearity under the studied ranges, with coefficients of determination (*R*^2^) above 0.99 in all matrices (Table 1).

LODs and LOQs were established as the mycotoxin concentration whose analytical signal is 3 and 10 times higher than noise signal, respectively. The signal-to-noise ratios were obtained from the quantitative ions of each mycotoxin, specified in Table S2. As can be seen in Table 1, LOQ values allowed the determination of mycotoxins at very low concentrations, especially in lung, achieving LOQ values of 0.001 ng g^*−*1^ for ENNB and BEA, and 0.015 ng g^*−*1^ for ENNB_1_. The lowest quantifiable concentration of ENNA_1_ was reached in brain (0.027 ng g^*−*1^) and 0.033 ng g^*−*1^ was the LOQ of ENNA in fat.

Repeatability and intermediate precision were calculated for all matrices to evaluate the precision of the proposed method. Each tissue was fortified at 25 and 5 ng g^*−*1^ of BEAs and ENNs in triplicate and analyzed in duplicate on the same day (intraday) and on non-consecutive 3 days (interday). Results were expressed as relative standard deviation (RSD) values (Table 2). The achieved values were suitable for all matrices not exceeding 9.8% of RSD.

In addition, trueness (expressed as apparent recovery) was calculated as the measured concentration divided by the actual concentration, in percentage. Each matrix was spiked at two concentrations (25 and 5 ng g^*−*1^) to assess a low and an intermediate concentration in triplicate. Recovery ranges were from 75 to 115% at 25 ng g^*−*1^ and from 82 to 118% at 5 ng g^*−*1^, with RSD values lower than 8.2%.

### Evaluation of emerging mycotoxin distribution in human

The application of the DLLME HPLC–MS/MS methodology allowed us to study and assess the incidence of emerging mycotoxins in human tissues, with the objective of evaluating its bioaccumulation and distribution. With this purpose, six different human forensic samples (brain, fat, heart, lung, liver, and kidney) from 26 autopsies were analyzed. A summary of the results is shown in Table 3. In addition, Figure S1 shows an example of the chromatographic separation obtained in real samples, specifically from a brain sample, in which all five mycotoxins studied were quantified.

**Table 3:** Summary of the occurrence of mycotoxins in different human tissues

		ENNB	BEA	ENNB_1_	ENNA_1_	ENNA
Brain	Mean ± SD^1^ (ng g^−1^)	0.7 ± 0.6^a^	0.1 ± 0.3^b^	0.2 ± 0.2 ^a,b^	0.2 ± 0.2^b^	0.1 ± 0.1^a,b^
	Range (ng g^−1^)	ND^2^–1.2	ND–0.70	ND–0.47	ND–0.46	ND–0.30
	Incidence (%)	15/26 (57.7)	4/26 (15.4)	12/26 (46.2)	14/26 (53.8)	9/26 (34.6)
	0.25th quantile (ng g^−1^)	0	0	0	0	0
	Median (ng g^−1^)	1.1	0	0	0.40	0
	0.75th quantile (ng g^−1^)	1.1	0	0.47	0.40	0.26
Liver	Mean ± SD (ng g^−1^)	7 ± 13^a^	NQ^3,b^	1.3 ± 1.0^a,c^	0.7 ± 0.6^b,d^	0.2 ± 0.4^c,d^
	Range (ng g^−1^)	0.47–56.1	ND–NQ	ND–4.0	ND–1.8	ND–1.0
	Incidence (%)	25/25 (100.0)	1/25 (4.0)	22/25 (88.0)	15/25 (60.0)	4/25 (16.0)
	0.25th quantile (ng g^−1^)	0.77	0	0.95	0	0
	Median (ng g^−1^)	2.3	0	1.0	0.89	0
	0.75th quantile (ng g^−1^)	5.2	0	1.2	0.96	0
Lung	Mean ± SD (ng g^−1^)	0.8 ± 0.3^a^	0.4 ± 0.4^b,c^	0.5 ± 0.3^c^	0.6 ± 0.3^a,b^	0.6 ± 0.3^b,c^
	Range (ng g^−1^)	ND–1.3	ND–0.9	ND–0.9	ND–0.9	ND–1.0
	Incidence (%)	24/26 (92.3)	13/26 (50.0)	21/26 (80.8)	22/26 (84.6)	21/26 (80.8)
	0.25th quantile (ng g^−1^)	0.78	0	0.61	0.64	0.72
	Median (ng g^−1^)	0.83	0.33	0.62	0.64	0.73
	0.75th quantile (ng g^−1^)	0.87	0.73	0.69	0.69	0.78
Kidney	Mean ± SD (ng g^−1^)	0.8 ± 0.4^a^	0.1 ± 0.3^b^	0.4 ± 0.5^b^	0.3 ± 0.4^b^	0.2 ± 0.3^b^
	Range (ng g^−1^)	ND–1.7	ND–1.5	ND–1.5	ND–1.2	ND–1.1
	Incidence (%)	21/26 (80.7)	1/26 (3.8)	11/26 (42.3)	11/26 (42.3)	6/26 (23.1)
	0.25th quantile (ng g^−1^)	0.80	0	0	0	0
	Median (ng g^−1^)	0.83	0	0	0	0
	0.75th quantile (ng g^−1^)	0.93	0	0.99	0.61	0
Heart	Mean ± SD (ng g^−1^)	NQ^a^	0.1 ± 0.3^a^	NQ^a^	NQ^a^	0.03 ± 0.12^a^
	Range (ng g^−1^)	ND–NQ	ND–1.3	ND–NQ	ND–NQ	ND–0.6
	Incidence (%)	16/25 (64.0)	8/25 (32.0)	7/25 (28.0)	8/25 (32.0)	6/25 (24.0)
	0.25th quantile (ng g^−1^)	0	0	0	0	0
	Median (ng g^−1^)	0.01	0	0	0	0
	0.75th quantile (ng g^−1^)	0.01	0	0.02	0.02	0
Fat	Mean ± SD (ng g^−1^)	0.2 ± 0.8^a^	0.5 ± 1.1^b^	1.8 ± 8.6^b^	1.0 ± 4.8^a^	4.7 ± 22.9^a^
	Range (ng g^−1^)	NQ–3.8	ND–3.9	ND–42.0	ND–23.5	ND–112.4
	Incidence (%)	24/24 (100.0)	22/24 (91.7)	23/24 (95.8)	23/24 (95.8)	20/24 (83.3)
	0.25th quantile (ng g^−1^)	0.01	0.03	0.02	0.01	0.01
	Median (ng g^−1^)	0.01	0.03	0.02	0.01	0.01
	0.75th quantile (ng g^−1^)	0.01	0.03	0.02	0.01	0.01

^1^*SD*, standard deviation

^2^*ND*, not detected

^3^*NQ*, not quantified

^a,b,c,d^The superscripts “a”, “b”, “c”, and “d” mean the classification into different groups for a specific compound as a result of Kruskal–Wallis ANOVA and Tukey test

Kruskal–Wallis one-way analysis of variance (ANOVA) on ranks and Tukey tests were performed to discriminate among the median mycotoxin concentrations in the different tissues. ENNB showed the highest incidence in all tissues and was detected in 100% of liver and fat samples with a mean concentration of 7 ± 13 ng g^*−*1^ and 0.2 ± 0.8 ng g^*−*1^, respectively. The occurrence of ENNB in kidney was significantly different from other mycotoxins, being classified in a different group. The highest incidence of all mycotoxins together was observed in fat, although in most cases the concentrations were below the LOQ. One forensic case in particular stands out as showing a significantly high concentration of ENNB_1_ (42 ng g^*−*1^), ENNA_1_ (23.5 ng g^*−*1^), and ENNA (112.4 ng g^*−*1^). Lung samples also showed a high incidence, but low accumulation was observed, as concentrations were below 0.8 ng g^*−*1^ in all cases.

Mycotoxins were statistically classified into several groups in lung, liver, brain, and fat, within which there were no statistically significant differences. Heart was the tissue with the lowest accumulation of mycotoxins with concentrations of ENNB, ENNB_1_, and ENNA_1_ below the LOQ. Thus, no significant differences among mycotoxins were found in this tissue.

**Fig. 3:**
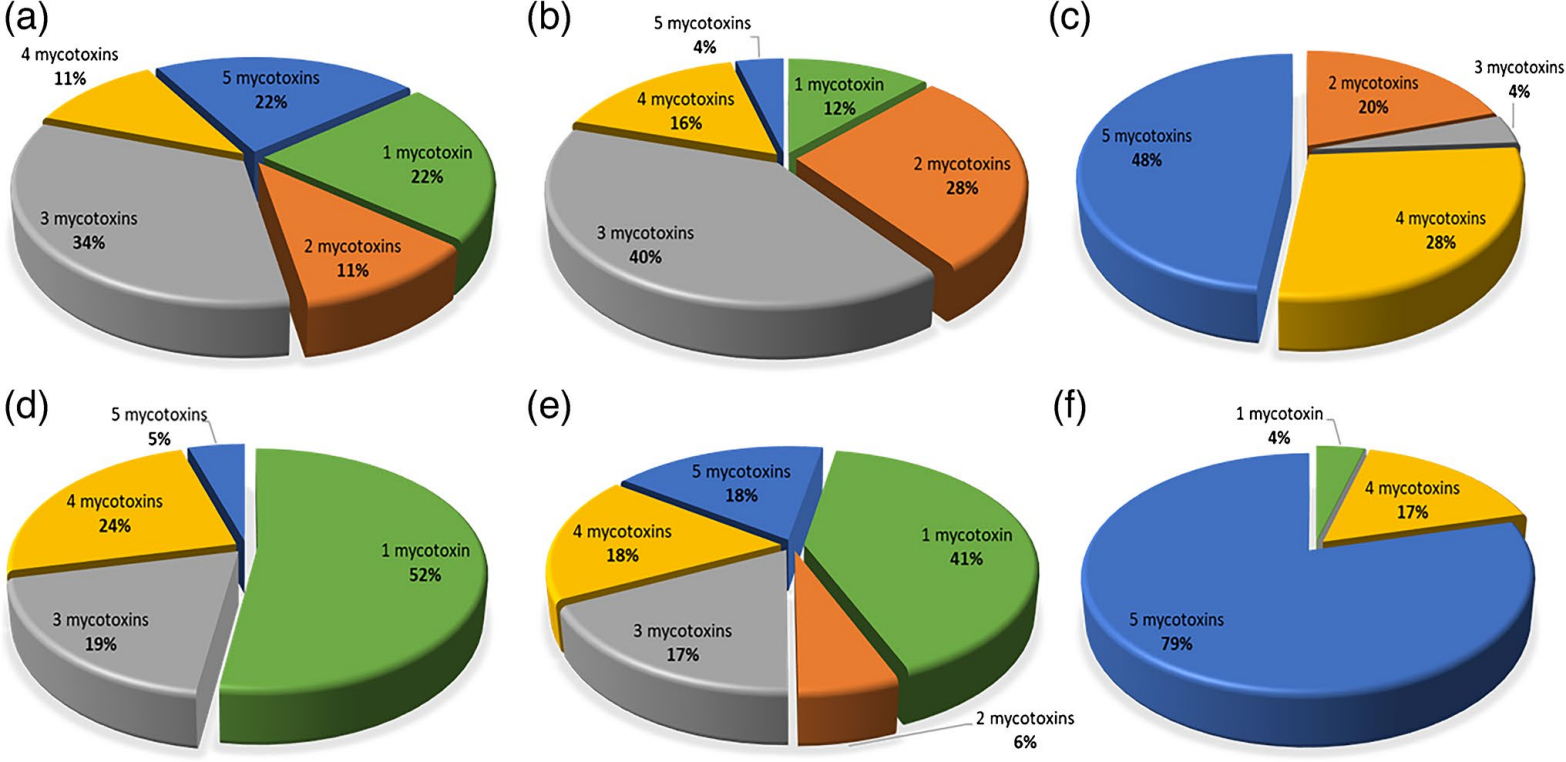
Co-occurrence of emerging mycotoxins in human tissues: brain (**a**), liver (**b**), lung (**c**), kidney (**d**), heart (**e**), and fat (**f**)

Furthermore, Table S3 shows the co-occurrence of the emerging mycotoxins depending on the tissue. The presence of only one mycotoxin, particularly ENNB, predominates in heart and kidney, with a percentage of frequency of 35.3% and 47.6%, respectively. The co-occurrence of three mycotoxins was most frequent in liver and brain samples. Specifically, the combination of ENNB, ENNB_1_, and ENNA_1_ was predominant in these tissues with a percentage of 40.0% and 22.2%, respectively. The set of the five mycotoxins was determined most frequently in lung (48.0%), in fat (79.2%), and also in brain at the same frequency of the combination of mycotoxins mentioned above (22.2%). Figure 3 summarizes the frequency of mycotoxin co-occurrence for each matrix. These results reveal a high co-occurrence rate of emerging mycotoxins in the different matrices. The multiple determination of mycotoxins by the proposed analytical method allows to study their presence and accumulation in different tissues and will allow a more accurate evaluation of their possible health risk.

**Fig. 4:**
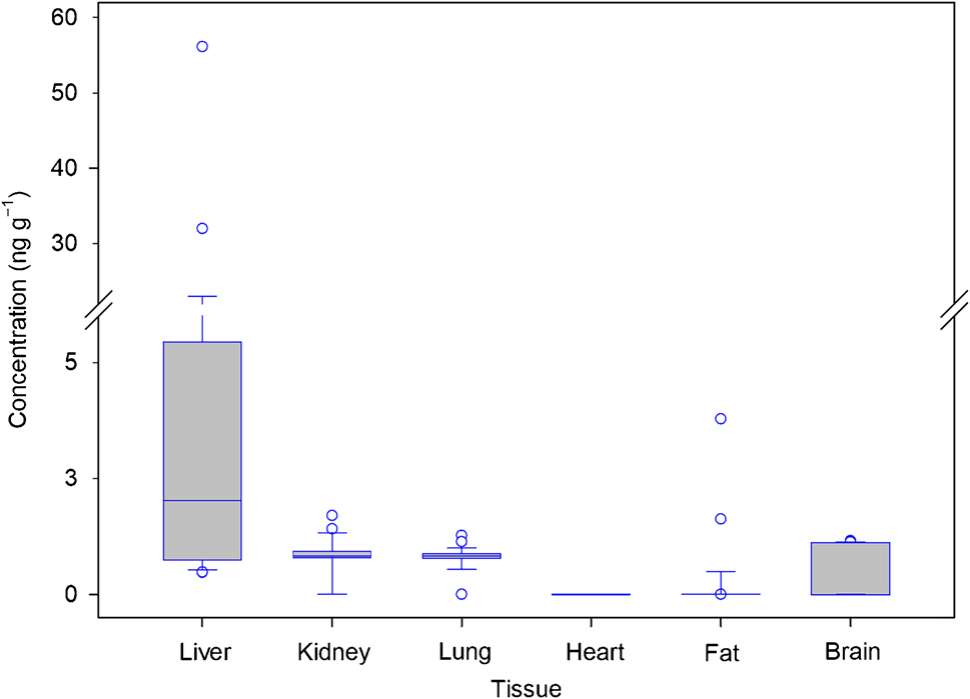
Box plot obtained after applying one-way analysis of variance test to concentration of ENNB in the different human tissues

After evaluating the incidence of emerging mycotoxins in each tissue, the concentration of each mycotoxin was compared in the different tissues, in order to consider their distribution in the human body. Statistical tests based on Kruskal–Wallis ANOVA and Dunn’s test were also applied. Significant differences were obtained for the median content of ENNB in liver compared to the accumulation found in heart (*p*-value < 0.001), fat (*p*-value < 0.001), and brain (*p* = 0.007). In addition, ENNB content in kidney and lung was significantly different from the concentrations found in heart and fat. Figure 4 shows the statistical box plot obtained for ENNB. As can be seen, liver showed the largest interquartile range, while the rest of the tissues present a low dispersion of data. The content of ENNB in liver showed a positive asymmetry, indicating a clustering of the data in the lower part of the distribution. In addition, outliers were observed above the upper limit, with the highest concentration being 56.1 ng g^*−*1^.

Significant differences were also obtained between the tissues for BEA, ENNA, ENNA_1_, and ENNB_1_. The incidence of BEA in human tissues was higher in fat and lung. Thus, the statistical analysis showed differences between its fat content and the rest of the tissues, except for lung: liver, kidney, and brain (*p*-value < 0.001), and heart (*p*-value = 0.003). ENNB_1_ concentration in liver was statistically different from brain, heart, kidney, and fat (*p*-value < 0.001). ENNA showed a higher occurrence in lung than in the rest of the tissues, and significant differences were observed between lung and heart (*p*-value < 0.001), brain (*p*-value = 0.007), and kidney (*p*-value = 0.008). Finally, significant differences were found between ENNB_1_ concentration in lung and in heart, liver, kidney, and brain (*p*-value < 0.001).

As a summary of the results obtained on the bioaccumulation of emerging mycotoxins in different human tissues, ENNB was the mycotoxin with the highest incidence in the human body, while BEA was the least frequently detected. ENNB tended to accumulate in liver, reaching higher concentrations than in the rest of the tissues, being frequent the combination of ENNB + ENNB_1_ + ENNA_1_ in this tissue. Fat and lung showed a high incidence of all emerging mycotoxins, with frequent co-occurrence of all five mycotoxins in the samples; however, the concentrations were very low, especially in fat where most of the concentrations were below LOQ. Emerging mycotoxins were detected in heart, but ENNB, ENNB_1_, and ENNA_1_ were not quantifiable. In case of kidney, the concentrations of ENNB in the samples were also significant, being the most frequent the presence of only this mycotoxin in the tissue. Finally, the co-occurrence of ENNB + ENNB_1_ + ENNA_1_ and the set of all five mycotoxins were frequent in brain samples. In this case, no significant differences were found between the different mycotoxins.

**Fig. 5:**
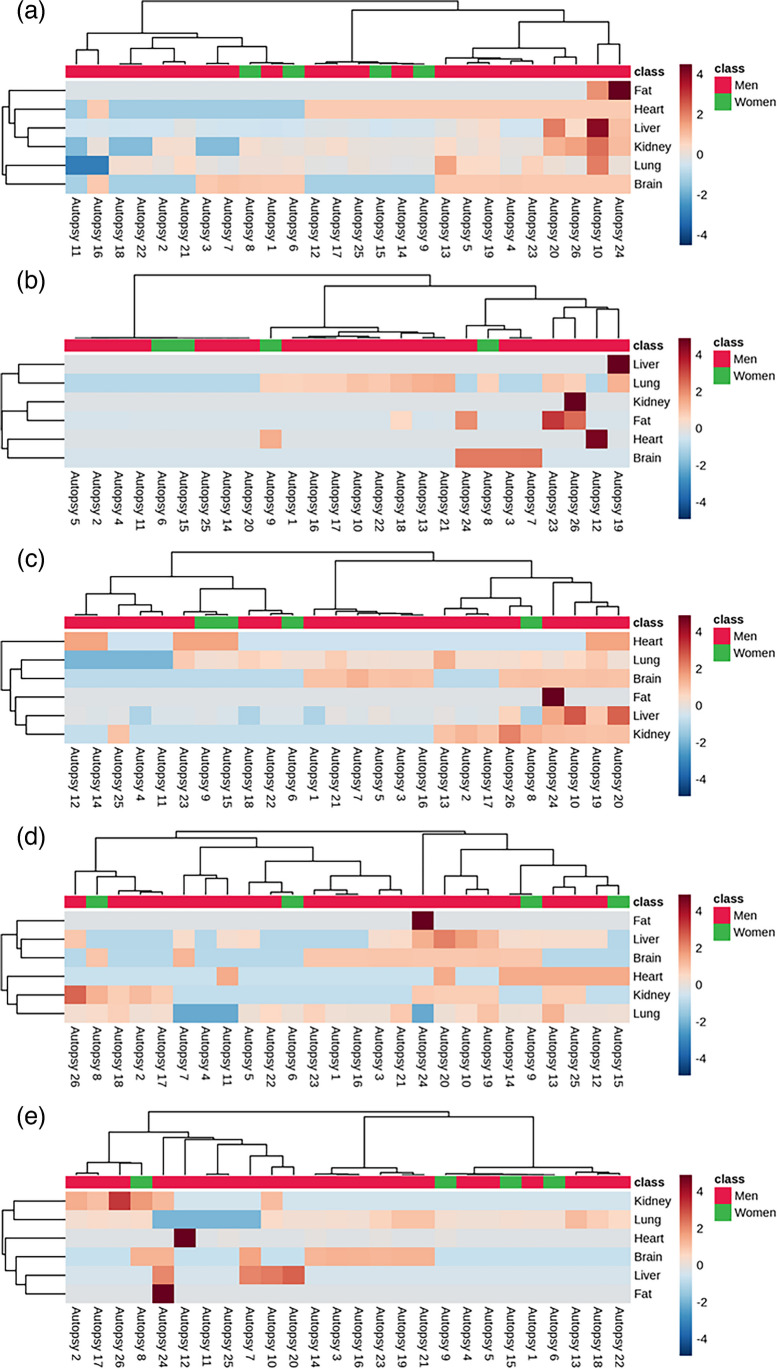
Hierarchical clustering heatmaps on the bioaccumulation of emerging mycotoxins in human tissues: ENNB (**a**), BEA (**b**), ENNB_1_ (**c**), ENNA_1_ (**d**), and ENNA (**e**)

In addition, hierarchical clustering heatmaps were performed in order to study the bioaccumulation of each emerging mycotoxin in the different human tissues representing the results of each forensic case used in this study. These graphs enable to observe unusually high or low values for the set of forensic cases and to establish associations between samples and tissues based on specific patterns. As can be observed in Fig. 5, particular cases exhibit outliers far from the mean values. One case is “Autopsy 24”, which showed an accumulation higher than average of all mycotoxins in fat. An opposite case is “Autopsy 11” which showed a content of ENNB, ENNB_1_, ENNA_1_, and ENNA below the average in lung.

Although the number of men and women in this study was not homogeneous (22 men and 4 women), the Mann–Whitney *U* test was applied between the two groups in order to explore possible differences in the concentration of each mycotoxin in the human body. In all cases, there were no statistically significant differences between men and women: ENNB (*p* = 0.416), BEA (*p* = 0.724), ENNB_1_ (*p* = 0.679), ENNA (*p* = 0.506), and ENNA_1_ (*p* = 0.714).

## Conclusion

The proposed methodology based on DLLME HPLC–MS/MS is presented as a valuable tool for the determination of ENNs and BEA in different human tissues in order to study their distribution in the human body. The analysis of liver, lung, brain, fat, kidney, and heart samples from 26 forensic autopsies has allowed a complete study of the distribution and accumulation of emerging mycotoxins for the first time. The proposed methodology achieved the quantification of these mycotoxins at very low concentrations. ENNB was the mycotoxin with the highest occurrence in the human body, accumulating mainly in liver, and being frequent the combination of ENNB + ENNB_1_ + ENNA_1_ in this tissue. On the contrary, BEA was the least frequently detected mycotoxin. Lung and fat showed a high co-occurrence of all five mycotoxins at low concentrations, while heart was the tissue with the lowest bioaccumulation, with occurrence of ENNB, ENNB_1_, and ENNA_1_ below the LOQs. Some particular forensic cases showed mycotoxin concentrations distant from the average values. Finally, no significant differences were found in mycotoxin bioaccumulation between men and women.

### Supplementary Information

Below is the link to the electronic supplementary material.

Supplementary file1 (DOCX 103 KB)

## Data Availability

Not applicable.
