# Supplementary material for: Evaluation of distribution of emerging mycotoxins in human tissues: applications of dispersive liquid–liquid microextraction and liquid chromatography-mass spectrometry
Source: Anal Bioanal Chem. 2023 Nov 21;416(2):449–59. doi: 10.1007/s00216-023-05040-8 (PMC10761373; doi:10.1007/s00216-023-05040-8)
Supplement: Supplementary file 1 — Supplementary file1 (DOCX 103 KB) [file 216_2023_5040_MOESM1_ESM.docx]

**Table S1.** Clinical data of autopsies

| Autopsy | Sex^a^ | Age | Type of death | Cause of death |
| --- | --- | --- | --- | --- |
| 1 | M | 32 | Suicide | Suffocation by hanging |
| 2 | M | 80 | Home accident | Traumatic brain injury and subdural hemorrhage |
| 3 | M | 71 | Suicide | Asphyxia secondary to hanging |
| 4 | M | 38 | Suicide | Suffocation by hanging |
| 5 | M | 29 | Suicide | Suffocation by hanging |
| 6 | W | 57 | Suicide | Inhalation of carbon monoxide |
| 7 | M | 43 | Natural death | Dilated cardiomyopathy |
| 8 | W | 75 | Traffic accident | Traumatic shock |
| 9 | W | 65 | Natural death | Under study |
| 10 | M | 75 | Natural death | Hypertensive heart disease |
| 11 | M | 82 | Suicide | Suffocation by hanging |
| 12 | M | 52 | Suicide | Suffocation by hanging |
| 13 | M | 64 | Natural death | Ischemic heart disease |
| 14 | M | 52 | Natural death | Generalised atheromatosis |
| 15 | W | 65 | Natural death | Coronary thrombosis |
| 16 | M | 83 | Accidental | Suffocation by hanging |
| 17 | M | 42 | Traffic accident | Polytrauma |
| 18 | M | 29 | Natural death | Gastrointestinal hemorrhage |
| 19 | M | 78 | Suicide | Traumatic brain injury |
| 20 | M | 49 | Traffic accident | Traumatic shock |
| 21 | M | 38 | Natural death | Cardiorespiratory arrest |
| 22 | M | 86 | Suicide | Suffocation by hanging |
| 23 | M | 65 | Natural death | Dilated cardiomyopathy |
| 24 | M | 57 | Natural death | Cardiorespiratory arrest |
| 25 | M | 82 | Natural death | Gastrointestinal hemorrhage. Hypovolemic shock |
| 26 | M | 65 | Agricultural machine accident | Traumatic brain injury |

^a^ Men (M), Woman (W)

**Table S2**. HPLC-QqQ-MS/MS conditions for the emerging mycotoxin determination

| Analyte | RT^a^ (min) | Molecular formula | Precursor ion (*m/z*) | Molecular ion | Product ion^b^ |
| --- | --- | --- | --- | --- | --- |
| ENNB | 6.88 | C_33_H_57_N_3_O_9_ | 657.50 | [M+NH_4_]^+^ | 196 (Q) |
|  |  |  |  |  | 214 (q) |
| BEA | 7.16 | C_45_H_57_N_3_O_9_ | 801.50 | [M+NH_4_]^+^ | 244 (Q) |
|  |  |  |  |  | 262 (q) |
| ENNB_1_ | 7.27 | C_34_H_59_N_3_O_9_ | 671.60 | [M+NH_4_]^+^ | 196 (Q) |
|  |  |  |  |  | 214 (q) |
| ENNA_1_ | 7.64 | C_35_H_61_N_3_O_9_ | 685.60 | [M+NH_4_]^+^ | 210 (Q) |
|  |  |  |  |  | 228 (q) |
| ENNA | 7.94 | C_36_H_63_N_3_O_6_ | 699.60 | [M+NH_4_]^+^ | 210 (Q) |
|  |  |  |  |  | 228 (q) |

^a^ RT: retention time
^b^ (Q) quantitative ion, (q) qualitative ion

**
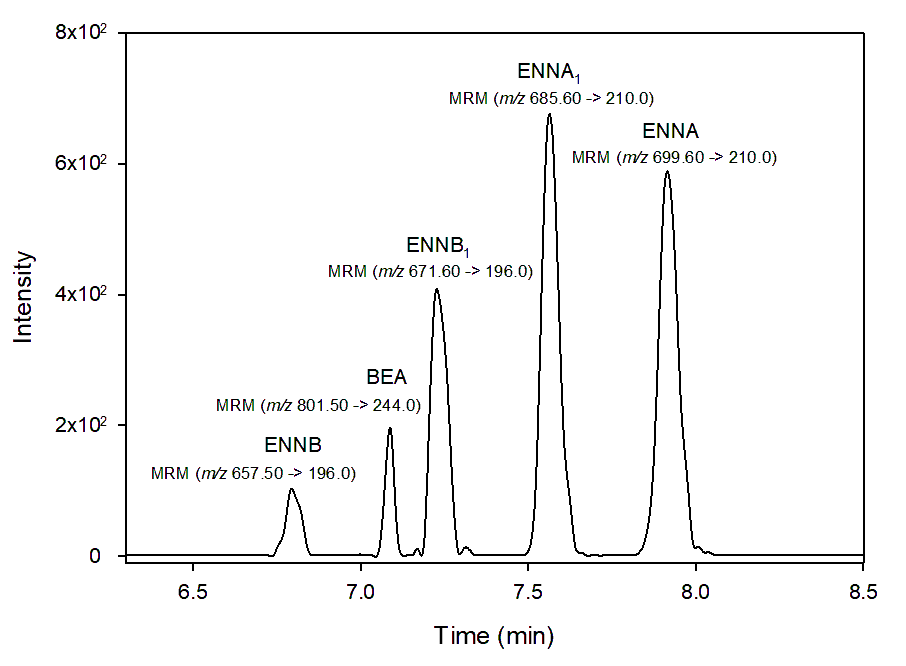
**

**Figure S1.** Chromatographic separation of enniatins and beauvericin in a brain human sample. Mycotoxin concentrations quantified in the sample: ENNB (1.1 ng g^-1^), BEA (0.7 ng g^-1^), ENNB_1_ (0.5 ng g^-1^), ENNA_1_ (0.4 ng g^-1^) and ENNA (0.3 ng g^-1^)

**Table S3.** Co-occurrence of enniatins and beauvericin in human tissues

|  | Co-occurrence (cases) | Mycotoxins | Frequency (%) |
| --- | --- | --- | --- |
| Liver | 1 mycotoxin (3/25) | ENNB | 3 (12.0) |
|  | 2 mycotoxins (7/25) | ENNB, ENNB_1_ | 7 (28.0) |
|  | 3 mycotoxins (10/25) | ENNB, ENNB_1_, ENNA_1_ | 10 (40.0) |
|  | 4 mycotoxins (5/25) | ENNB, ENNB_1_, ENNA_1_, ENNA | 4 (16.0) |
|  |  | ENNB, BEA, ENNB_1_, ENNA_1_ | 1 (4.0) |
| Kidney | 1 mycotoxin (11/21) | ENNB | 10 (47.6) |
|  |  | ENNA_1_ | 1 (4.8) |
|  | 3 mycotoxins (4/21) | ENNB, ENNB_1_, ENNA_1_ | 4 (19.1) |
|  | 4 mycotoxins (5/21) | ENNB, ENNB_1_, ENNA_1_, ENNA | 5 (23.8) |
|  | 5 mycotoxins (1/21) | ENNB, BEA, ENNB_1_, ENNA_1_, ENNA | 1 (4.8) |
| Lung | 2 mycotoxins (5/25) | ENNB, ENNA_1_ | 2 (8.0) |
|  |  | ENNB, ENNA | 1 (4.0) |
|  |  | ENNB, ENNB_1_ | 2 (8.0) |
|  | 3 mycotoxins (1/25) | ENNB, ENNA_1_, ENNA | 1 (4.0) |
|  | 4 mycotoxins (7/25) | ENNB, ENNB_1_, ENNA_1_, ENNA | 6 (24.0) |
|  |  | BEA, ENNB_1_, ENNA_1_, ENNA | 1 (4.0) |
|  | 5 mycotoxins (12/25) | ENNB, BEA, ENNB_1_, ENNA_1_, ENNA | 12 (48.0) |
| Heart | 1 mycotoxin (7/17) | ENNB | 6 (35.3) |
|  |  | ENNA_1_ | 1 (5.9) |
|  | 2 mycotoxins (1/17) | ENNB, ENNA | 1 (5.9) |
|  | 3 mycotoxins (3/17) | ENNB, BEA, ENNB_1_ | 2 (11.8) |
|  |  | ENNB, ENNB_1_, ENNA | 1 (5.9) |
|  | 4 mycotoxins (3/17) | ENNB, BEA, ENNA_1_, ENNB_1_ | 2 (11.8) |
|  |  | ENNB, BEA, ENNA_1_, ENNA | 1 (5.9) |
|  | 5 mycotoxins (3/17) | ENNB, BEA, ENNB_1_, ENNA_1_, ENNA | 3 (17.6) |
| Fat | 1 mycotoxin (1/24) | ENNB | 1 (4.2) |
|  | 4 mycotoxins (4/24) | ENNB, BEA, ENNB_1_, ENNA_1_ | 3 (12.5) |
|  |  | ENNB, ENNB_1_, ENNA_1_, ENNA | 1 (4.2) |
|  | 5 mycotoxins (19/24) | ENNB, BEA, ENNB_1_, ENNA_1_, ENNA | 19 (79.2) |
| Brain | 1 mycotoxin (4/18) | ENNB | 3 (16.7) |
|  |  | ENNA_1_ | 1 (5.6) |
|  | 2 mycotoxins (2/18) | ENNB, ENNB_1_ | 1 (5.6) |
|  |  | ENNA_1_, ENNA | 1 (5.6) |
|  | 3 mycotoxins (6/18) | ENNB, ENNA_1_, ENNA | 1 (5.6) |
|  |  | ENNB, ENNB_1_, ENNA_1_ | 4 (22.2) |
|  |  | ENNB_1_, ENNA_1_, ENNA | 1 (5.6) |
|  | 4 mycotoxins (2/18) | ENNB, ENNB_1_, ENNA_1_, ENNA | 2 (11.1) |
|  | 5 mycotoxins (4/18) | ENNB, BEA, ENNB_1_, ENNA_1_, ENNA | 4 (22.2) |
